# Documenting Environmental Contamination in Vulnerable Populations

**DOI:** 10.5696/2156-9614-11.31.210901

**Published:** 2021-08-17

**Authors:** Sandra Page-Cook

**Affiliations:** *Journal of Health and Pollution*, Pure Earth, New York, USA

*The Journal of Health and Pollution* (*JH&P*) was conceived in 2010, publishing the first issue in February 2011. Our original impetus to create *JH&P* was to increase understanding of the health effects of environmental contamination in low- and middle-income countries (LMIC). This problem was thought to be large and not well understood, with mortality and morbidity in LMIC much greater than that in high-income countries (HIC), even after accounting for the compounding effects of poverty, malnutrition, overcrowding and poor housing conditions.^1^ In 2010 there was little published research documenting concrete findings, and little originating from affected LMIC.

Our scientific focus was point source pollution, related health impacts, environmental control and remediation technologies, and ambient and indoor pollution, particularly heavy metals, pesticides, radionuclides, dioxins, polychlorinated biphenyls (PCBs), polycyclic aromatic hydrocarbons (PAHs), volatile organic compounds (VOCs) and air particulates. This remained the main focus, but we also published articles on medical, pharmaceutical and solid waste in LMIC, many articles on the intersection between our contaminants of focus and occupational health, and several sets of conference abstracts, including those from the last three meetings of the International Network on Children's Health, Environment and Safety (INCHES).^2^ The health effects and remediation of lead (Pb) remained a constant theme, with about one third of all articles touching on Pb.^3^ In the past year, *JH&P* along with most other health related journals received submissions focusing on the SARS-CoV-2 pandemic as it related to our areas of focus.

We quickly developed an additional goal of nurturing authors from underrepresented areas of the world, typically LMIC. In addition to living with high amounts of environmental contamination, researchers from many LMIC face the same pressure to publish as their colleagues in HIC, but often resources such as information, training, translation, editing and support networks are not available.^4^

To support authors we aimed to provide detailed and high quality reviews to all articles within our area of focus, created a small grants program to support unfinished work (2012–2016), and partnered with AuthorAID^5^ to deliver an online course in Research Writing to Environmental Health Researchers, limited to researchers from countries within World Health Organization (WHO) Mortality Strata B through E.^6^ We also had no charges for authors or readers, supporting *JH&P* with funds from the World Bank and the United States Agency for International Development (USAID), and provided free English language copyediting to all accepted manuscripts.

We are proud to have published articles from countries very underrepresented in the scientific literature, including Algeria,^7^ Armenia,^8^ Belize,^9^ Botswana,^10^ Jamaica,^11^–^14^ Liberia,^15^ Mongolia,^16^ Myanmar/Burma,^17^,^18^ Nepal,^19^,^20^ Sri Lanka,^21^ Sudan,^22^,^23^ Tajikistan,^24^ Tanzania,^25^–^27^ Togo,^28^, ^29^ Uganda^30^–^33^ and Zimbabwe.^34^

Submission and acceptance data for the thirteen most represented countries by corresponding author can be found in Table 1.

**Table 1:** Article Submissions and Publication Rate by Country

**Country of corresponding author**	**Number of submissions**	**Number of articles published**	**Publication rate**
**Nigeria**	213	85	39.9%
**India**	82	28	34.1%
**United States**	41	24	58.5%
**Ghana**	31	17	54.8%
**Iran**	28	0	0.0%
**Indonesia**	24	5	20.8%
**Bangladesh**	23	8	34.8%
**Philippines**	21	11	52.4%
**Morocco**	18	5	27.8%
**Iraq**	17	4	23.5%
**Egypt**	13	5	38.5%
**Kenya**	12	6	50.0%
**Ethiopia**	12	8	66.7%

Language consistently presented the greatest challenge. For some of our authors, English was a third or even fourth language, and sometimes barriers to communication in complex peer reviews could not be surmounted. Additionally, we were not able to provide any article processing (reviews, editing) in any language other than English, leading to Latin America being underrepresented in our submission data. Acceptance and publication rates tended to be highest in countries where the population is educated in English. In recent years we received some well written and interesting submissions from Iran, however were unable to process them due to continuing sanctions. Our acceptance rate was 22% in 2020.

By 2018, *JH&P* was indexed in the Directory of Open Access Journals (DOAJ), Scopus, PubMed, Clarivate Analytics Emerging Sources Citation Index, Biological Abstracts and BIOSIS Previews and was a member of the Committee on Publication Ethics (COPE).

We developed a robust Editorial Board and attracted over 700 peer reviewers—too many to individually mention and thank here. Many reviewers went out of their way to provide detailed, constructive and kind reviews to under-supported authors, and for that I am very grateful.

This is the final edition of *JH&P* with Pure Earth^35^ as the publisher. I am proud of our achievements and hope we have enhanced the knowledge bank of environmental contamination challenges globally.
